# Promising Treatment for Type 2 Diabetes: Fecal Microbiota Transplantation Reverses Insulin Resistance and Impaired Islets

**DOI:** 10.3389/fcimb.2019.00455

**Published:** 2020-01-17

**Authors:** Hui Wang, Yuan Lu, Yan Yan, Shanshan Tian, Dongjie Zheng, Dongjing Leng, Cao Wang, Jingfeng Jiao, Zhiguo Wang, Yunlong Bai

**Affiliations:** ^1^Department of Pharmacology (State-Province Key Laboratories of Biomedicine-Pharmaceutics of China, Key Laboratory of Cardiovascular Medicine Research, Ministry of Education), College of Pharmacy, Harbin Medical University, Harbin, China; ^2^Translational Medicine Research and Cooperation Center of Northern China, Chronic Disease Research Institute, Heilongjiang Academy of Medical Sciences, Harbin, China

**Keywords:** diabetic, microbiota, FMT, apoptosis, inflammation

## Abstract

Type 2 diabetes is a common metabolic disorder related to insulin resistance, or deficiency of insulin secretion, caused by decreased insulin sensitivity and destruction of islet structure and function. As the second human genome, the microbiota has been observed to have a growing relationship with diabetes in recent years. Microbiota imbalance has been hypothesized to be involved in the regulation of energy metabolism and the inflammatory immune response in diabetes. The present study aimed to investigate whether fecal microbiota transplantation (FMT) could alleviate the symptoms associated with type 2 diabetes. To this end, a type 2 diabetes mouse model was first established through the consumption of a high-fat diet combined with streptozotocin (100 mg/kg), and FMT was used to rebuild the gut microbiota of diabetic mice. Fasting blood glucose, oral glucose tolerance tests, and HbA1c levels were monitored, while the hypoglycemic effects of FMT were also observed. Insulin levels were tested by ELISA and related indexes such as HOMA-IR, HOMA-IS, and HOMA-β were calculated. We found that insulin resistance and pancreatic islet β-cells were improved after FMT treatment. Meanwhile, the markers of inflammation in the pancreatic tissue were detected by ELISA and immunohistochemistry, which indicated that inflammatory response decreased following FMT treatment. Furthermore, flow cytometry and western blot results revealed that FMT inhibited the β-cell apoptosis. Here, the effect of FMT on hypoglycemia in type 2 diabetes was addressed by improving insulin resistance and repairing impaired islets, thereby providing a potential treatment strategy for type 2 diabetes.

## Introduction

Type 2 diabetes mellitus (T2DM) is the most common endocrine and metabolic disease, which is characterized by high blood glucose, insulin resistance, and a relative deficiency of insulin. Clinical epidemiological data show that the number of patients with T2DM is increasing rapidly worldwide; the number is estimated to exceed 439 million by 2030 (Shaw et al., 2010). Sustained hyperglycemia and metabolic disorders can lead to tissues and organs dysfunction, particularly the eyes, kidneys, cardiovascular and nervous systems (Koopal et al., 2018). Presently, the main causes of T2DM are peripheral insulin resistance and pancreatic islet β-cell injury and dysfunction, which leads to abnormal blood glucose regulation and serious complications (Ruiz et al., 2018; Sun et al., 2018). However, the currently available drugs, diet control, insulin injections, and other treatments do not prevent the occurrence of diabetes or the development of various chronic complications.

The intestinal microflora is a general term for the various microorganisms that are located in the intestinal environment (Kerr et al., 2015). The number of intestinal microorganisms is more than 10^14^/mL in the human body, which forms a complex and relatively independent micro-ecosystem; microorganisms participate in numerous functions such as substance transformation and energy metabolism (Jia et al., 2017). When the ratio of beneficial bacteria to harmful bacteria is disturbed, the balance of microbiota is broken, and metabolic disorders occur in the human body, leading to other related diseases. It is believed that T2DM is the result of impaired islet β- cells function due to genetic and environmental factors. Owing to in-depth research on intestinal function, the role of microbiota in the progression of type 2 diabetes is becoming increasingly clear and acknowledged (Kang et al., 2018). Studies have shown that intestinal microflora is significantly different between patients with type 2 diabetes and healthy individuals (Savilahti et al., 2018). The proportion of *Firmicutes* and *Clostridium* was significantly reduced in T2DM patients, and the levels of *Bacteroides, Escherichia coli* and other conditional pathogenic bacteria were increased (Larsen et al., 2010; Qin et al., 2012; Wang et al., 2018). In contrast, probiotics have been shown to improve intestinal microflora disturbances and effectively alleviate the symptoms of diabetes. Due to the complexity of the intestinal microflora, its structure and function have not yet been fully understood yet, and its specific role in T2DM has not been completely elucidated.

Apoptosis is the programmed cell death that occurs in multicellular organisms (Curti et al., 2017). Previous studies have shown that impaired islet β-cell function is associated with decreased β-cells due to apoptosis (Butler et al., 2003; Curti et al., 2017). Imbalanced intestinal microflora activates low-grade chronic inflammation of islets, which may cause damage and dysfunction of islet β-cells and promote β-cell apoptosis. In the current study, we aimed to evaluate whether fecal microbiota transplantation (FMT) could alleviate the symptoms in a high-fat diet (HFD) and streptozotocin-induced diabetes. Our results showed that rebuilding gut microbiota could be decreased fasting blood glucose (FBG) levels and improves insulin resistance in T2DM mice by relieving islets destruction. From the perspective of gut microbiota, our study aimed to support the theory that remodeling intestinal microflora could improve T2DM, which elucidates the capability of FMT to control diabetes mellitus and offer a new consideration for FMT as a valuable treatment strategy in metabolic diseases.

## Materials and Methods

### Animals and Ethics Approval

Kunming (KM) mice, weighing 20–22 g, were provided by the Animal Center of the Second Affiliated Hospital of Harbin Medical University. The methods were performed in accordance with the National Guidelines for Experimental Animal Welfare (The Ministry of Science and Technology, People's Republic of China, 2006). All experimental protocols were pre-approved by the Experimental Animal Ethics Committee of Harbin Medical University, China (No. HMUIRB 20180025).

Type 2 diabetes was induced by a high-fat diet for 6 weeks and combined with a single intraperitoneal injection of streptozotocin (100 mg/kg) (Sigma, St. Louis, MO) dissolved in 0.01 mol/L citric acid solution (pH = 4.3). One week later, FBG was measured through a tail vein. T2DM mouse models were selected in which the FBG value was higher than 11.1 mmol/L. The mice were randomly divided into control, T2DM, and T2DM+FMT groups. All groups were raised under standard temperature (23 ± 1°C), humidity (55% ± 5%), and a normal diet for 8 weeks. Healthy KM mice (22–25 g) with FBG lower than 6.0 mmol/L were randomly selected as the normal control group. Mice in the T2DM+FMT group were intragastrically administered with 0.3 ml fecal suspension daily for 8 weeks; the control and T2DM mice received equivalent volumes of sterilized distilled water. The FBG and OGTTs levels were observed during the experiment in all groups.

### Fecal Microbiota Transplantation (FMT)

To serve as donor mice, KM mice were obtained and housed under constant temperature and humidity conditions for 1 week. Animals were placed in an empty autoclaved cage a day before collecting the fecal samples. The mice is defecated normally, and the first three fecal pellets of each animal were collected in an empty 1.5 ml tube using a sterile toothpick. The tube is quickly closed, placed in liquid nitrogen, and then transferred to a refrigerator at −80°C for storage until preparation of the fecal suspension (Ericsson et al., 2017). Fecal samples (300 mg) were dissolved in 10 ml sterilized distilled water and mixed well for use.

### ELISA

Blood samples were collected from the medial canthus vein of each mouse. Pancreas tissue homogenate was obtained by sonication. Plasma HbA1c and serum insulin were measured using the mouse ELISA Kit (Cloud-Clone Corp, Wuhan, China) after the blood samples were centrifuged (1,000 × g) for 15 min at 4°C. Pancreas tissue IL-6, IL-10, and TNF-α were detected using the mouse ELISA Kit (Cloud-Clone Corp, Wuhan, China). The ELISAs were performed according to the manufacturer's protocols.

### Hematoxylin and Eosin (HE) Staining

Mice were administered 2% Tribromoethanol (0.01 ml/g) by intraperitoneal injection. After anesthetizing, pancreatic tissue was removed and immediately immobilized for 24 h in 4% paraformaldehyde. Following fixation, the tissue was processed by dehydration with a gradient concentration of ethanol, cleared in xylene, embedded in paraffin, and sliced at 4 μm thickness. HE staining was performed according to the manufacturer's protocols (Solarbio, Beijing, China) (He et al., 2018).

### Immunohistochemistry (IHC)

Tissue sections were deparaffinized and antigen retrieval is performed using the pressure cooker method. The sections were blocked with normal sheep serum at room temperature for 30 min, followed by incubation at 4°C overnight with primary antibodies for IL-6, IL-10, TNF-α (All antibody, Bioss, Beijing, China, 1:100). The sections were washed three times with PBS and then incubated with secondary antibodies (ZSGB, Beijing, China) at 37°C for 1 h. DAB solution was added to cover tissue sections and incubated at room temperature for 2 min. Hematoxylin stained for 15 s, dehydrated, transparent and mounted for paraffin section. A “quick score” method for IHC semiquantitation validation was used. The score range is 0–18 that is positively correlated to the positive intensity and the expression quantity. The formula is: Quick Score = A _the percentage of positive cells_ × B _the average intensity_. A is assigned into 0 to 6 (1 = 0–4%; 2 = 5–19%; 3 = 20–39%; 4 = 40–59%; 5 = 60–79%; 6 = 80–100%). B is assigned into negative, weak, intermediate and strong staining that is represented by 0, 1, 2, and 3, respectively.

### Western Blot

Total protein was extracted from the pancreatic tissue and separated by electrophoresis using 12% sodium dodecyl sulfate (SDS) polyacrylamide gels. The proteins are transferred to polyvinylidene difluoride membranes (Millipore, Bedford, MA, USA). The membranes were blocked with 4% milk for 2 h and then incubated at 4°C overnight with primary antibodies for Bcl-2, Bax, Caspase-3, and GAPDH (Bcl-2, SANTA CRUZ, 1:1000; Bax, proteintech, 1:1000; Caspase-3, Millipore, 1:200; GAPDH, ZSGB, Beijing, China, 1:1000). The membranes were washed three times with PBS containing 0.5% Tween 20 (PBS-T) and then incubated with secondary antibodies (ZSGB, Beijing, China, 1:10000) at room temperature for 1 h. The images are captured on Odyssey 1.2 software (LI-COR Biosciences, Valencia, CA). Relative band densities were quantified using Image Studio software with GAPDH as an internal control protein.

### Flow Cytometry

Pancreatic islet single-cell suspensions are prepared from the pancreatic tissue through a sieve. The cells were washed three times with p0hosphate-buffered saline (PBS), centrifuged (1,000 × g) for 5 min at 4°C, and the cell concentration is maintained at approximately 1 × 10^5^ cells/ml. The flow cytometry is performed with the Annexin V-FITC Apoptosis Detection Kit according to the manufacturer's protocols (Vazyme, Nanjing, China). The cells were suspended in 100 μl 1× Binding Buffer solution, stained with 5 μl Annexin V-FITC and 5 μl PI Staining Solution, and incubated at room temperature for 10 min. Next, 400 μl 1× Binding Buffer is added and apoptosis is assessed by flow cytometry (BD LSRFortessaTM, Franklin Lakes, NJ, USA).

### 16S Sequencing and Bioinformatics Analysis

First, the fecal samples of each mouse are collected in a sterilized tube. The samples are stored at −80°C refrigerator until use. The genomic DNA of each fecal sample was extracted based on the SDS method. The purity and concentration of DNA are detected by agarose gel electrophoresis and the samples are then diluted to 1 ng/μL with sterile water. Using the diluted genomic DNA as the template, the V3–V4 region is amplified by the enzyme and PCR buffer (New England Biolabs, Massachusetts, USA). According to the concentration of the PCR products, the samples were mixed and purified on the 2% agarose gel electrophoresis, and then target bands were cut and recycled by GeneJET Kit (Thermo Scientific, Waltham, USA). Libraries are constructed by the Ion Plus Fragment Library Kit 48 rxns (Thermo Scientific, Waltham, USA) and finally the raw data were obtained according to manufacturer's protocols of Ion S5^TM^XL (Thermo Scientific, Waltham, USA). Cluster analysis of OTU is performed using Uparse software (Uparse v7.0.1001, http://drive5.com/uparse/) (Edgar, 2013) and alpha diversity is calculated using Qiime software.

### Statistical Analysis

All measurement data were presented as mean ± SEM and analyzed by GraphPad Prism 5. For normal distribution data, differences between groups are analyzed by One-way variance analysis (ANOVA). For non-normal distribution data, we performed a Kruskal-Wallis analysis. *P* < 0.05 was considered to indicate a statistically significant difference.

## Results

### FMT Alleviated Hyperglycemia in T2DM Mice

**Figure 1:**
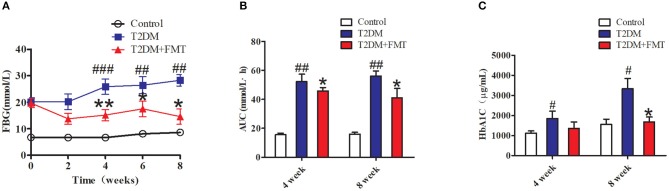
The effect of FMT on hyperglycemia inT2DM mice. **(A)** Relative levels of FBG on different groups during the experiment period. **(B)** Effect on the areas under the curve of glucose (AUC). Mice were administrated 20% glucose (2 g/kg) and blood glucose was tested in 0, 30, 60, 120 min. AUC = 1/2 × [0.5 × (FBG_0_ + FBG_30_) + 0.5 × (FBG_30_ + FBG_60_) + (FBG_60_ + FBG_120_)]. **(C)** Effect on plasma HbA1c level was detected by Elisa *P < 0.05 vs. T2DM, **P < 0.01 vs. T2DM, ^#^*P* < 0.05 vs. Control, ^##^*P* < 0.01 vs. Control, ^###^*P* < 0.001 vs. Control, *n* = 4–6.

To explore the therapeutic effects of FMT against hyperglycemia in T2DM mice, fecal samples or normal saline were administered to mice for 8 weeks. We first characterized the changes in microbiota that occurred after FMT using 16S sequencing. According to the OTUs clustering analysis results, the species number was significantly increased after FMT treatment for 8 weeks as shown in Figure S1A. Concurrently, the Alpha Diversity of Shannon index and Simpson index indicated that the distribution of intestinal flora had a certain recovery in the T2DM+FMT group (Figures S1B,C). The above results showed that after FMT treatment, the intestinal flora of T2DM mice was more uniformly. The blood glucose level was monitored every 2 weeks until the eighth week. As shown in Figure 1A, the control group and T2DM+FMT groups had stable FBG levels stable during the experiment period, while the T2DM group was sustained hyperglycemia. Compared with the T2DM group, the FBG level in the T2DM+FMT group declined from the second week and was significantly reduced by the fourth week after FMT treatment. Meanwhile, the OGTTs were tested and the areas under the curve of glucose were calculated. It was observed that OGTTs also improved from the fourth week in the T2DM+FMT group (Figure 1B). We also collected blood samples and detected plasma HbA1c levels during the fourth and eighth weeks, and expectedly, the HbA1c level decreased in the FMT group over time (Kruskal-Wallis test, *p* < 0.05) (Figure 1C). These results suggest that FMT could stablish and decrease blood glucose and improve glucose tolerance.

### FMT Improved Insulin Resistance and Repaired Injured Islets

**Figure 2:**
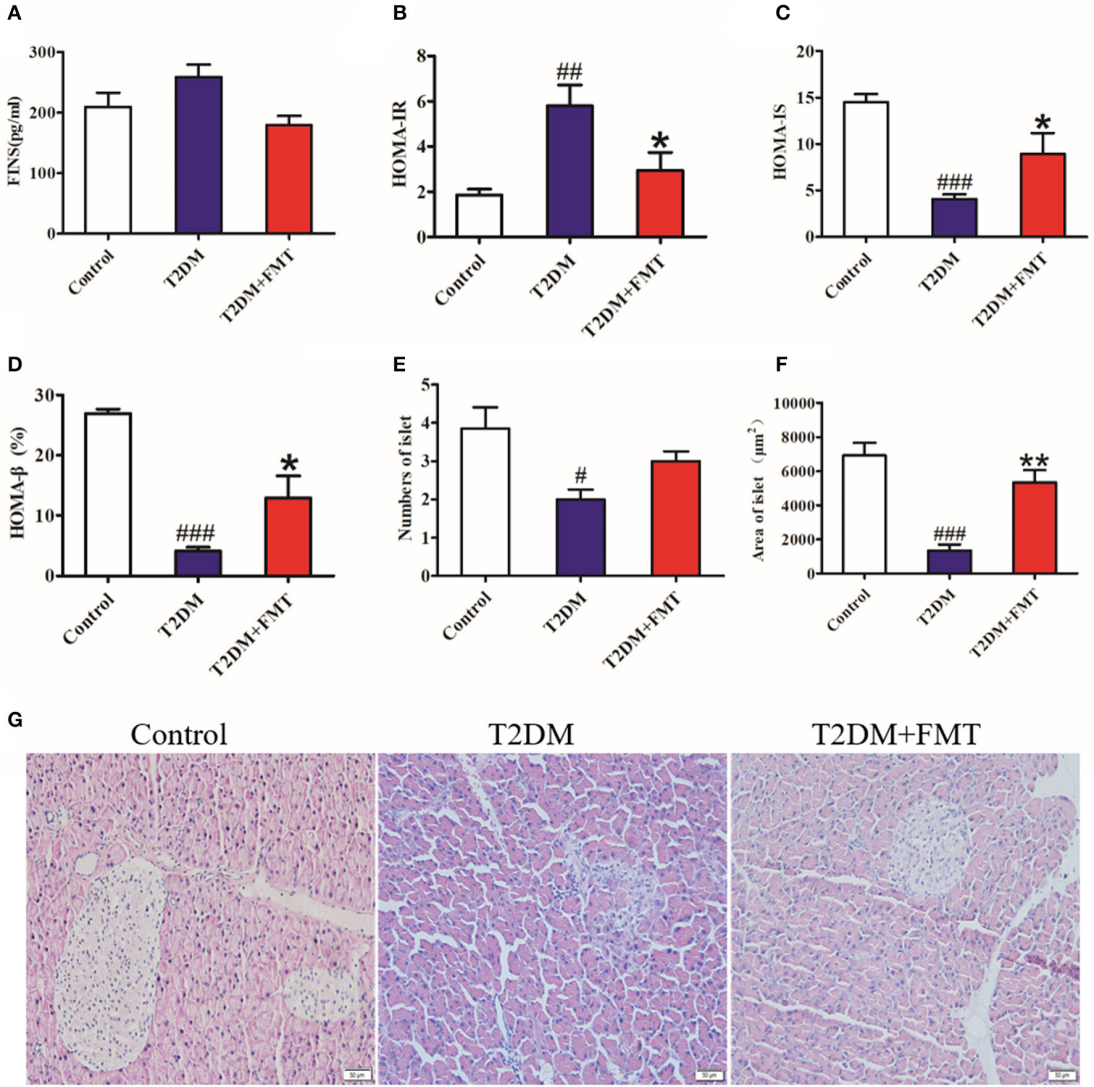
The effect of FMT on insulin resistance and islet of the pancreas. **(A)** Fasting serum insulin level in different group. **(B)** HOMA-IR index in different group. HOMA-IR index = FBG × FINS/22.5 **(C)** HOMA-IS index in different group. HOMA-IS index = 1/HOMA-IR. **(D)** HOMA-β index in different group. HOMA-β index = 20 × FINS/(FBG-3.5) (%) **(E)** Relative amount of the islet in each group observed by HE staining image. **(F)** Relative area of the islet in each group observed by HE staining image. **(G)** Representative HE images of pancreas. *P < 0.05 vs. T2DM, **P < 0.01 vs. T2DM, ^#^*P* < 0.05 vs. Control, ^##^*P* < 0.01 vs. Control, ^###^*P* < 0.001 vs. Control, *n* = 4–6.

Since insulin resistance is the main cause of T2DM (Fraulob et al., 2010), the insulin-related indicators were tested after 8 weeks of treatment. Blood samples were obtained from the medial canthus vein and the serum insulin levels were measured using an ELISA kit. As shown in Figure 2A, the serum insulin level increased in the T2DM group compared with the control group, and it was reversed following FMT treatment. We also calculated the index of insulin resistance and insulin sensitivity. The T2DM group had the highest index of HOMA-IR and lowest HOMA-IS, but the T2DM+FMT group had largely ameliorated insulin resistance and increased insulin sensitivity (Figures 2B,C). Type 2 diabetes is characterized by insulin resistance, reduced insulin sensitivity, and impaired pancreatic β-cell function. To identify the effect of FMT on the function of islets, the HOMA-β and pancreas histology was performed. The results of HOMA-β indicated that the FMT group had greatly reversed pancreatic β-cell function compared with the T2DM group (Figure 2D). Simultaneously, HE staining revealed that the islets of the T2DM group were seriously damaged; however, the number and the size of islets was obviously increased in the FMT group (Kruskal-Wallis test, *p* < 0.05) (Figures 2E–G). Thus, FMT could improve insulin resistance and alleviate islet damage.

### FMT Inhibited Chronic Inflammation in Pancreatic Tissue

**Figure 3:**
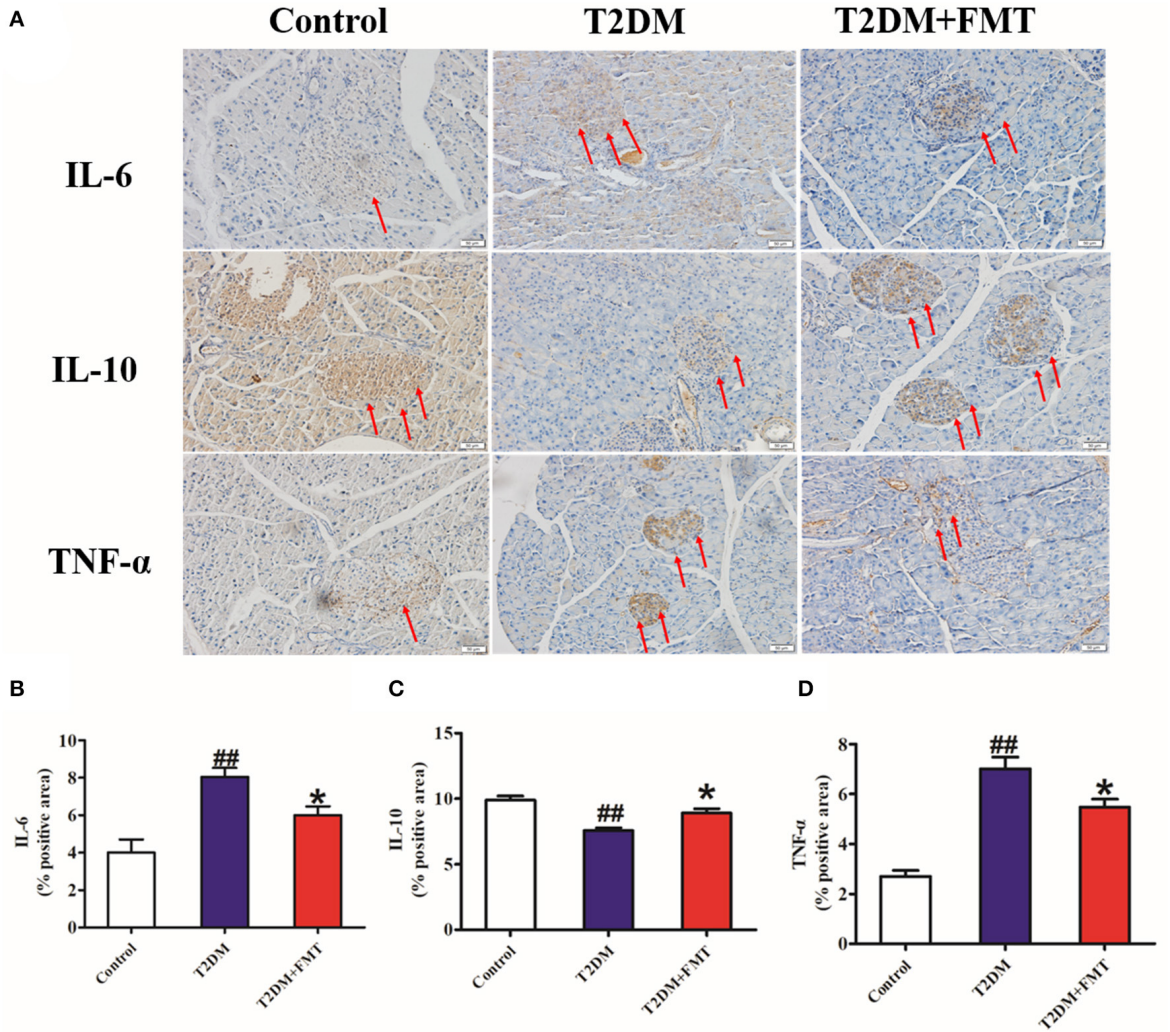
The effect of FMT on pro-inflammatory and anti-inflammatory cytokines. **(A)** Representative IHC images of IL-6, IL-10, and TNF-α in pancreas. **(B)** The positive expression rate of IL-6 in pancreas detected by IHC. **(C)** The positive expression rate of IL-10 in pancreas detected by IHC. **(D)** The positive expression rate of TNF-α in pancreas detected by IHC. *P < 0.05 vs. T2DM, ^##^*P* < 0.01 vs. Control, *n* = 8.

**Figure 4:**
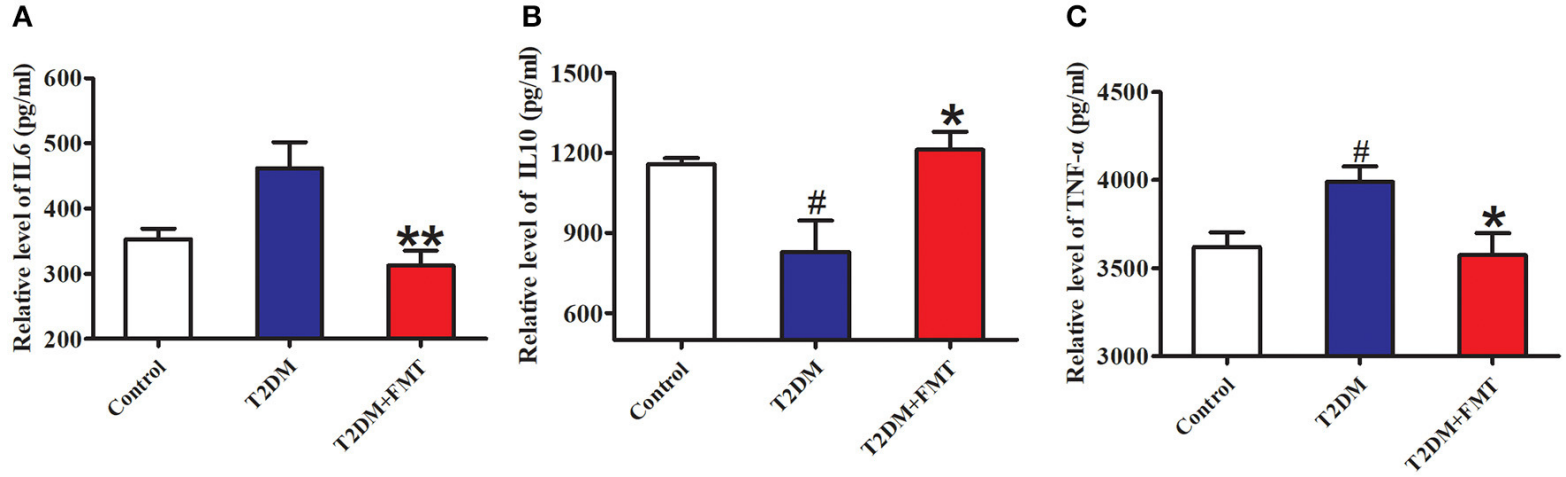
The content of pro-inflammatory and anti-inflammatory cytokines. **(A)** The content of IL-6 in pancreas detected by ELISA. **(B)** The content of IL-10 in pancreas detected by ELISA. **(C)** The content of TNF-α in pancreas detected by ELISA. *P < 0.05 vs. T2DM, **P < 0.01 vs. T2DM, ^#^*P* < 0.05 vs. Control, *n* = 4–6.

Generally, secretion of pro-inflammatory cytokines such as tumor necrosis factor-alpha (TNF-α) (De Taeye et al., 2007) and interleukin-6 (IL-6) increases, or secretion of anti-inflammatory cytokines including interleukin-10 (IL-10) decreases, this causes chronic inflammation in tissues leading to islet structure damaged and dysfunction in diabetic patients. To elucidate the mechanism underlying FMT-induced improvement of islet structural damage, we further detected the expression level of pro-inflammatory cytokines and anti-inflammatory. After 8 weeks of blood glucose monitoring, mice were sacrificed and pancreas samples were stored for testing inflammatory cytokines. The quick score results of IHC shown that pro-inflammatory factors of IL-6 and TNF-α were concentrated in the range of 4–9, and anti-inflammatory of IL-10 was between 0 and 3 in the T2DM group. However, the score was reversed after FMT treatment (Tables S1–S3). IHC results indicated that IL-6 and TNF-α in the T2DM+FMT group were lower than in the T2DM group; IL-10 was much higher in the T2DM group (Figure 3). Moreover, the inflammatory cytokines in pancreatic tissues were also detected by ELISA. Similarly, the results were consistent with the IHC data (Kruskal-Wallis test, *p* < 0.05) (Figure 4). FMT may improve damaged islets through decreased the secretion of pro-inflammatory cytokines and increased secretion of anti-inflammatory cytokines.

### FMT Protected Islet Structure and Function by Decreasing Pancreatic β-Cell Apoptosis

**Figure 5:**
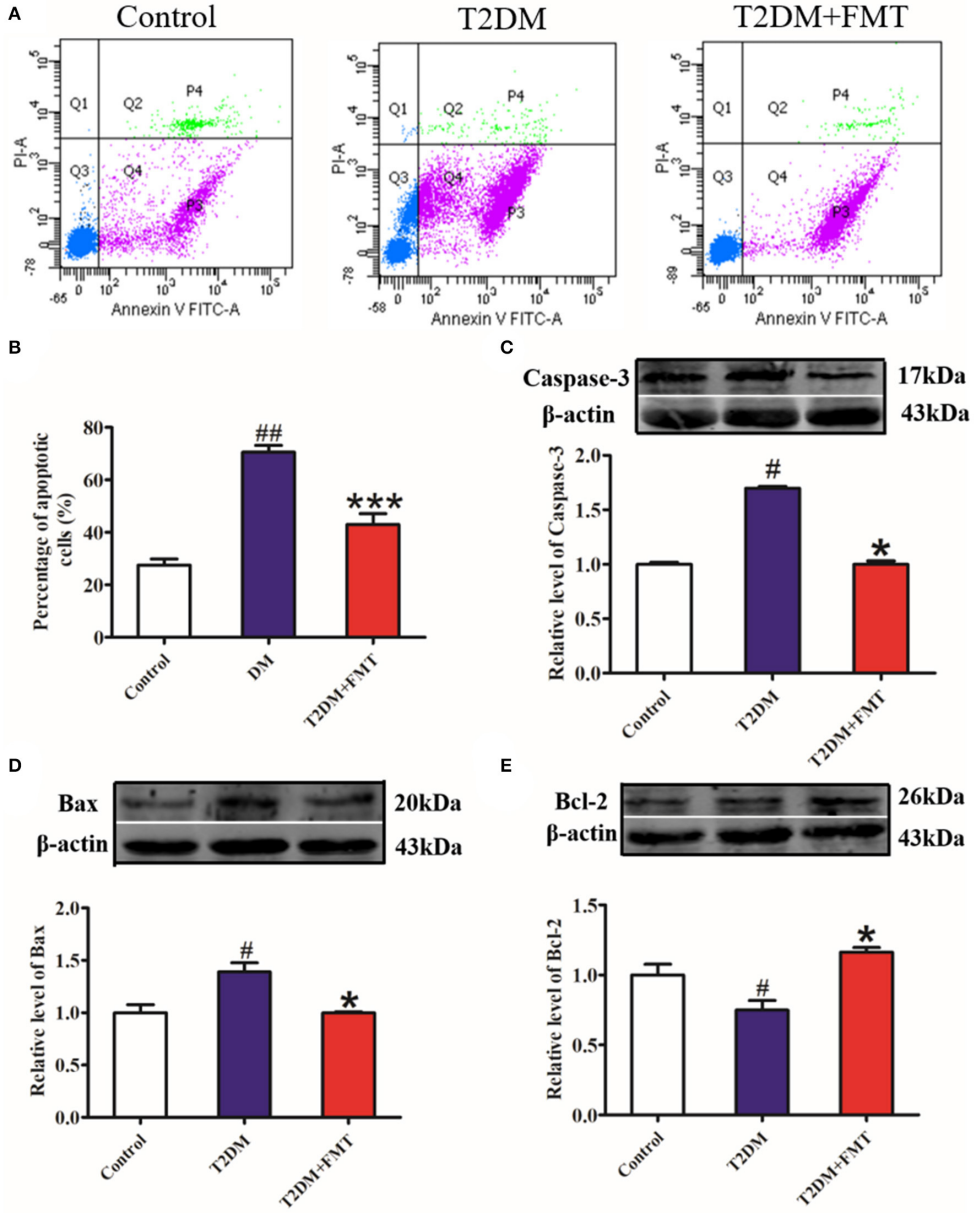
The effect of FMT on apoptosis in pancreas tissue. **(A)** Representative flow cytometry images for pancreatic islet single cell. **(B)** Relative percentage of apoptosis cells in pancreas tissue calculated by flow cytometry. **(C)** The expression level of cleaved Caspase-3 in pancreas detected by western blot. **(D)** The expression level of Bax in pancreas detected by western blot. **(E)** The expression level of Bcl-2 in pancreas detected by western blot. *P < 0.05 vs. T2DM, ***P < 0.001 vs. T2DM, ^#^*P* < 0.05 vs. Control, ^##^*P* < 0.01 vs. Control, *n* = 4–6.

The increase in β-cell apoptosis is a fundamental reason for impaired islet structure and function induced by the inflammatory response (Butler et al., 2003). To investigate whether FMT could protect islets by inhibiting pancreatic β-cell apoptosis, pancreatic islet single-cell suspensions were prepared from the pancreatic tissue and apoptosis was assessed by flow cytometry. The results showed that cell apoptosis in the T2DM group significantly increased, compared to the control group, whereas cell apoptosis was attenuated in the T2DM+FMT group (Figures 5A,B). Meanwhile, we also checked the expression levels of apoptosis-related indicators, including the pro-apoptotic proteins Caspase-3 and Bax, and anti-apoptotic protein Bcl-2. The western blotting results showed that the expression level of cleaved Caspase-3 and Bax were notably upregulated in the T2DM group compared with the control group and reversed by FMT (Figures 5C,D). The anti-apoptotic protein Bcl-2 was downregulated in the T2DM group and upregulated after treatment with FMT (Figure 5E). Hence, we drew the conclusion that diabetes promotes islet cell apoptosis, while FMT promotes islet cell regeneration by suppressing cell apoptosis.

## Discussion

Gut microbiota primarily colonizes in the host intestinal mucosa, and its effects are conferred to human pathophysiology and metabolism (Kau et al., 2011). Microflora disorder is a general characteristic found in diabetes and other metabolic diseases (Aydin et al., 2018). Numerous studies have shown that the disorder of gut microbiota has the potential to affect the progression of diabetes (Saad et al., 2016; Nie et al., 2019). It has been shown that patients with T2DM have a moderate dysbiosis between the butyrate-producing bacteria and *Lactobacillus* species (Qin et al., 2012). Furthermore, various metabolites produced by microbiota, such as short-chain fatty acids, were significantly different between type 2 diabetic and normal hosts (Salek et al., 2007). Many intervention studies have shown that the intake of intestinal probiotics could effectively improve intestine microecology disorder, and relieve the symptoms of diabetic patients.

A few data have demonstrated that the formation of gut microbiota could be reshaped to improve or control disease status (Shen et al., 2015). Fecal microbiota transfer (FMT) is a method to treat diseases by reconstructing the microbiota (Lee et al., 2018). There is increasing evidence regarding the treatment potential of FMT based on an already developed clinical plan that has been the first-line therapy for recurrent *Clostridium difficile* infections (Cammarota et al., 2017). However, understanding the influence of the intestinal microbiota on metabolic diseases is in the initial stage and the data regarding the function of FMT on type 2 diabetes are still scarce. In our study, we established T2DM animal models by feeding mice high-fat diet combined with streptozotocin and observed that FMT treatment successfully reduced FBG and improved glucose tolerance on diabetic mice (Figures 1A,B). It was possible to restore the balance of intestinal microflora to promote host homeostasis (Burrello et al., 2018). According to a recent study, in metabolic syndrome, when commensal bacteria from various phyla stay in a certain range, they contribute to control the ratio of pathogenic species and support optimal physical conditions (Burrello et al., 2018). Glycated hemoglobin test usually reflects the patient's blood glucose control in the last 8–10 weeks and our ELISA results of HbA1c further provided evidence of the hypoglycemic effect of FMT (Figure 1C). These results showed that FMT had a therapeutic effect on hyperglycemia. As reported in the literature, FMT could display a recovered phenotype upon transfer donor's intestinal flora to the receptor, which was consonant with our current experimental results (de Groot et al., 2017).

T2DM is characterized by insulin resistance, which has also been confirmed in our study (Figure 2B). Alterations of gut microbiota composition are highly linked with adiposity and insulin resistance, which has been considered to be an environmental factor for type 2 diabetes (Borody et al., 1989; de Groot et al., 2017). After T2DM mice reconstructed their microbiota through the feces received from normal mice, fasting insulin levels were decreased and insulin resistance was improved (Figures 2A,B). At the same time, the insulin sensitivity index (HOMA-IS) was increased in the T2DM+FMT group. Similarly, in a clinical trial, obese patients received FMT treatment from lean healthy individuals showed a positive change in insulin sensitivity (Aron-Wisnewsky et al., 2019). For the current research, dysbiosis of gut microbiota is associated with the development of insulin resistance and diabetes (de Groot et al., 2017). The mechanism underlying the gut microbiota-induced amelioration of insulin resistance may be through changes in body energy balance or by alleviating obesity caused by a high-fat diet. However, the exact mechanism needs to be explored comprehensively.

To the best of our knowledge, microbiota imbalance induces more gram-negative bacteria, which produce high amounts of LPS and activate low-grade chronic inflammation of the islets. When the microbiota of normal mice was transplanted into diabetic mice, the secretion of pro-inflammatory factors decreased and anti-inflammatory secretion increased in pancreatic tissues (Figures 3, 4). Studies have shown that FMT is involved in the low-grade inflammation characterized by metabolic disorders. It has been reported that therapeutic FMT could decrease the secretion of inflammatory factors and triggered several immune-mediated signal-pathways in colitis (Burrello et al., 2018). As mentioned in another study, transplantation of gut microbiota like *F. prausnitzii* prevented the inflammation damage in the pancreas (Ganesan et al., 2018). These findings were similar to our HE staining and HOMA-β index results, which indicated that the size of the islet and the function of pancreatic islet β cell was recovered after FMT treatment in diabetic mice (Figures 2D–G). IL-6 and TNF-α are pro-inflammatory cytokines with multiple functions that can directly act on islet cells, causing pancreatic islet β cell injuries (Park et al., 2018). Low-grade chronic inflammation caused by microbiota imbalance usually leads to the destruction of islet structure and impaired pancreatic islet β cell function, and islet β-cells apoptosis is the fundamental reason for the destruction of islet structure. Nonetheless, islet injury and dysfunction were reversed when the inflammatory responses and the apoptosis of islet beta cells were relieved through FMT treatment (Figure 5).

Although there are several studies about the function of FMT, the mechanism underlying FMT-induced alleviation of the disease remains largely unelucidated. Diabetes symptoms may be relieved through the synergistic effects between the commensal gut microbiota after FMT treatment, or possibly triggered by multiple immune-inflammatory processes and pathways. Future research should focus on the bacterial taxonomic and functional changes affiliated with FMT treatment in diabetic patients, as well as how FMT affects the metabolism of other organs in long-term improvement. Due to the complexity of intestinal flora, further research should explore whether the particular microflora species or communities in FMT make an effort on preventing and treating diabetes. This may provide a novel perspective and reference that could be verified in population-based studies about the effect of FMT on diabetes for the next work. Long-term restoration of gut microbiota through FMT may be used as a promising therapeutic application for diabetes.

In conclusion, this study demonstrated that high-fat diet induced T2DM can be treated through FMT by improving the insulin resistance and attenuating pancreatic islet β-cell destruction. We discovered that FMT could alleviate hyperglycemia, which provided a bacterial-based treatment strategy for the management of T2DM.

## Data Availability Statement

All datasets generated for this study are included in the article/Supplementary Material.

## Ethics Statement

The animal study was reviewed and approved by the Experimental Animal Ethics Committee of Harbin Medical University, China (No. HMUIRB 20150034).

## Author Contributions

HW conceptualized and designed the experiments in this study. YL, YY, ST, DL, and CW performed the experimental procedures related to the study. ST, DZ, and JJ revised the article. ZW proofread and discussed articles related to this research. YB provided the financial support and reviewed the experimental directions. All authors contributed to the generation of experimental data for this study.

### Conflict of Interest

The authors declare that the research was conducted in the absence of any commercial or financial relationships that could be construed as a potential conflict of interest.

## Abbreviations

AUC: Areas under the curve of glucose
FBG: Fasting blood glucose
FMT: Fecal microbiota transplantation
HE: Hematoxylin and eosin
HFD: High-fat diet
HOMA-IR: Homeostasis model assessment-Insulin resistance
HOMA-IS: Homeostasis model assessment-Insulin sensitive
HOMA-β: Homeostasis model assessment-β
IHC: Immunohistochemistry
IL-10: Interleukin-10
IL-6: Interleukin-6
OGTTs: Oral glucose tolerance tests
PBS: Phosphate-buffered saline
SDS: Sodium dodecyl sulfate
T2DM: Type 2 diabetes mellitus
TNF-α: Tumor necrosis factor-alpha.
