# Supplementary material for: Promising Treatment for Type 2 Diabetes: Fecal Microbiota Transplantation Reverses Insulin Resistance and Impaired Islets
Source: Front Cell Infect Microbiol. 2020 Jan 17;9:455. doi: 10.3389/fcimb.2019.00455 (PMC6979041; doi:10.3389/fcimb.2019.00455)
Supplement: Supplementary file 1 [file Data_Sheet_1.pdf]

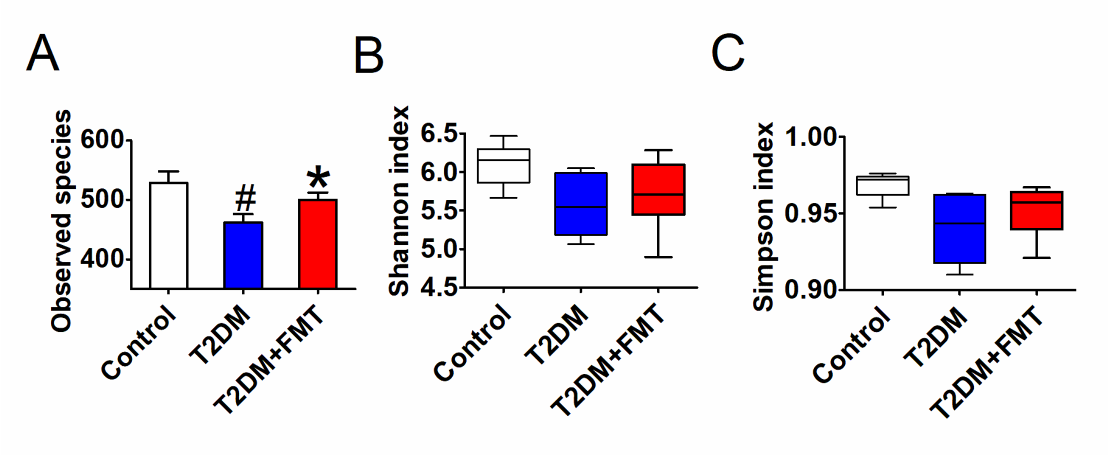

Fig.S1. The effect of FMT on gut microbiota inT2DM mice. **(A)** Observed species. **(B)** Box plot of Shannon index. **(C)** Box plot of Simpson index. *P < 0.05 versus T2DM, #P < 0.05 versus Control, n = 5.

Table S1 Multiplicative quick score of IL-6 in islets

	Quick score=A x B																		
	**0**	**1**	**2**	**3**	**4**	**5**	**6**	**7**	**8**	**9**	**10**	**11**	**12**	**13**	**14**	**15**	**16**	**17**	**18**
**Control**	10	4	3	0	1	0	0	0	0	0	0	0	0	0	0	0	0	0	0
**T2DM**	0	3	2	0	4	0	3	0	0	5	0	0	1	0	0	0	0	0	0
**T2DM+FMT**	9	3	0	0	4	0	2	0	0	0	0	0	0	0	0	0	0	0	0

Notes: A = the percentage of positive cells. B=the average of intensity.

Table S2 Multiplicative quicks core of IL-10 in islets

	Quick score=A x B																		
	**0**	**1**	**2**	**3**	**4**	**5**	**6**	**7**	**8**	**9**	**10**	**11**	**12**	**13**	**14**	**15**	**16**	**17**	**18**
**Control**	0	1	1	1	0	0	3	0	0	4	0	0	5	0	0	3	0	0	0
**T2DM**	7	5	3	2	0	0	0	0	0	1	0	0	0	0	0	0	0	0	0
**T2DM+FMT**	1	1	3	1	0	0	2	0	0	8	0	0	2	0	0	0	0	0	0

Notes: A = the percentage of positive cells. B=the average of intensity.

Table 3 Multiplicative quick score of TNF-α in islets

	Quick score=A x B																		
	**0**	**1**	**2**	**3**	**4**	**5**	**6**	**7**	**8**	**9**	**10**	**11**	**12**	**13**	**14**	**15**	**16**	**17**	**18**
**Control**	12	4	1	0	1	0	0	0	0	0	0	0	0	0	0	0	0	0	0
**T2DM**	1	1	3	0	4	0	4	0	0	5	0	0	0	0	0	0	0	0	0
**T2DM+FMT**	6	5	3	0	2	0	1	0	0	1	0	0	0	0	0	0	0	0	0

Notes: A = the percentage of positive cells. B=the average of intensity.
